# Bilateral adrenal infarction in pregnancy: a diagnostic challenge (a case report)

**DOI:** 10.11604/pamj.2026.53.166.52450

**Published:** 2026-04-20

**Authors:** Asma Ladib, Jihen Ladib, Hela Abdessalem, Bilel Ben Amor, Marouene Zeglaoui, Ines Mazhoud, Ali Jlali, Fethi Jebali, Lotfi Grati

**Affiliations:** 1 Department of Anesthesia and Intensive Care B, Maternity Center, Fattouma Bourguiba University Hospital, Monastir, Tunisia,; 2 Department of Pharmacy, Fattouma Bourguiba University Hospital, Monastir, Tunisia,; 3 Department of Endocrinology, Fattouma Bourguiba University Hospital, Monastir, Tunisia,; 4 Department of Radiology, Maternity Center, Fattouma Bourguiba University Hospital, Monastir, Tunisia

**Keywords:** Bilateral adrenal infarction, pregnancy, adrenal insufficiency, thrombosis, case report

## Abstract

Bilateral adrenal infarction in pregnancy is a rare but potentially life-threatening condition that can lead to acute adrenal insufficiency and is often misdiagnosed due to nonspecific symptoms. We report a 25-year-old primigravida at 34 weeks presenting with abdominal and lumbar pain, fever, and vomiting, initially treated as acute pyelonephritis. Clinical deterioration with tachypnea and metabolic acidosis prompted further investigations. Computed tomography pulmonary angiography ruled out pulmonary embolism, while contrast-enhanced abdominal computed tomography revealed bilateral non-enhancing adrenal enlargement. Serum cortisol was inappropriately low (128.2 nmol/L), supporting acute adrenal insufficiency. Immediate intravenous hydrocortisone and therapeutic anticoagulation led to rapid improvement. The patient delivered vaginally at 38 weeks under epidural analgesia with stress-dose corticosteroids. This case highlights the importance of early imaging and prompt corticosteroid therapy in pregnant patients with unexplained persistent abdominal pain.

## Introduction

Bilateral adrenal infarction during pregnancy is a rare but potentially life-threatening condition, most often related to adrenal vein thrombosis in the context of the physiological hypercoagulable state of gestation [1,2]. Its clinical presentation is frequently nonspecific and may mimic more common obstetric, infectious, surgical, or thromboembolic conditions, leading to diagnostic delay [3]. Early recognition is crucial, as delayed diagnosis can result in acute adrenal insufficiency with severe maternal consequences [4]. We report a case of bilateral adrenal infarction occurring in the third trimester of pregnancy, initially misdiagnosed as acute pyelonephritis, and we highlight the diagnostic challenges and multidisciplinary management that led to a favorable outcome.

## Methods

**Patient information:** a 25-year-old primigravida (G1P0) at 34 weeks and 4 days of gestation, with no significant past medical history and an otherwise uncomplicated pregnancy, was admitted for severe abdominal pain associated with left lumbar pain, fever (38.3°C), and persistent vomiting. There was no personal or family history of thromboembolic disease, and no prior medical or surgical interventions were reported.

**Timeline of current episode:** day 0: admission for abdominal and lumbar pain, fever, and vomiting; empirical antibiotic therapy (cefotaxime) initiated for suspected acute pyelonephritis. Day 2: clinical deterioration with persistent fever, intractable vomiting, and onset of dyspnea and marked tachypnea (46 breaths/min); transfer to intensive care unit. Day 2-3: computed tomography (CT) pulmonary angiography ruled out pulmonary embolism; contrast-enhanced abdominal CT demonstrated bilateral adrenal enlargement without contrast enhancement. Day 3: initiation of intravenous hydrocortisone (200 mg/day) and therapeutic anticoagulation (enoxaparin 0.6 mL twice daily). Day 5: clinical improvement; transfer back to the obstetrics department. At 38 weeks of gestation: premature rupture of membranes; enoxaparin discontinued 24 hours before induction; epidural analgesia performed; vaginal delivery with stress-dose hydrocortisone (100 mg IV bolus at onset of active labor). At 2.5 months postpartum: comprehensive thrombophilia work-up performed.

**Clinical findings:** on admission, the patient presented with fever (38.3°C), left lumbar tenderness with a positive Giordano sign, and vomiting. After 48 hours, her condition worsened with the onset of dyspnea, marked tachypnea (46 breaths/min), and tachycardia (110 beats/min), while remaining hemodynamically stable (blood pressure 120/80 mmHg). Physical examination revealed no signs of peritonitis or surgical abdomen.

**Figure 1:**
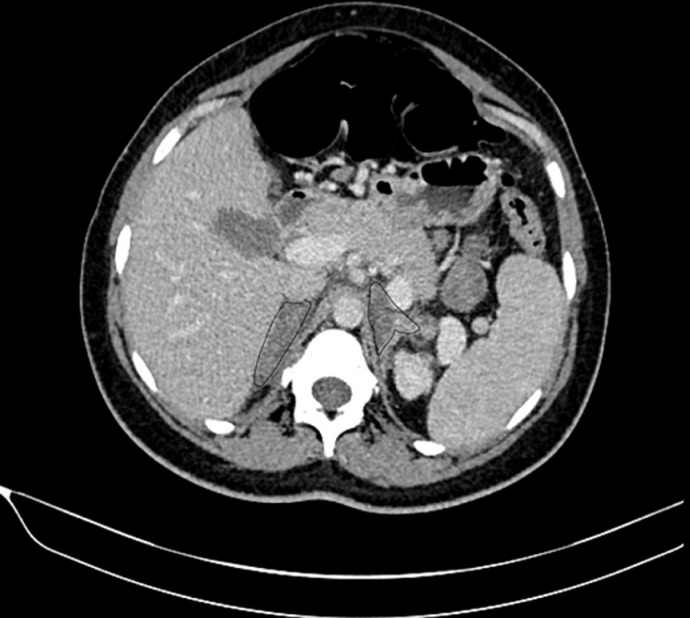
axial view of contrast-enhanced abdominopelvic computed tomography showing bilateral adrenal enlargement with lack of contrast enhancement in a 34-week pregnant patient with bilateral adrenal infarction

**Figure 2:**
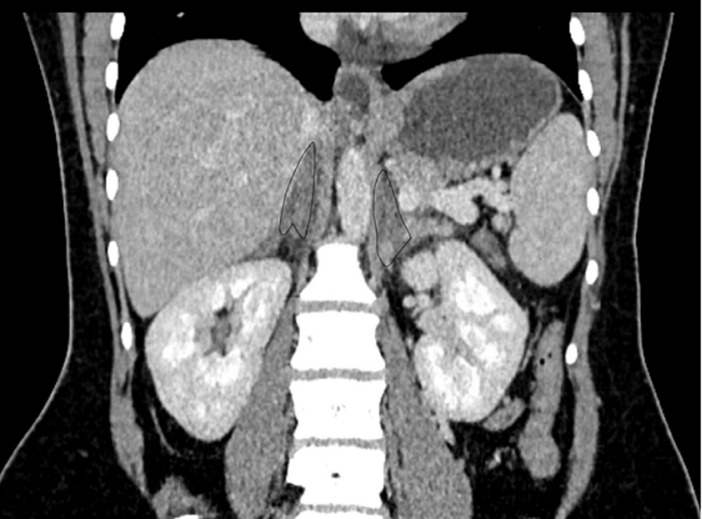
coronal view of contrast-enhanced abdominopelvic computed tomography showing bilateral adrenal gland enlargement with non-enhancing areas in a 34-week pregnant patient

**Diagnostic assessment:** initial abdominal ultrasound excluded surgical emergencies such as acute appendicitis, cholecystitis, or placental abruption. Urinalysis showed leukocyturia (20 cells/field) and hematuria (10 cells/field) without visible microorganisms; subsequent urine culture remained negative. Based on fever, lumbar tenderness, and leukocyturia, a diagnosis of acute pyelonephritis was suspected, and empirical cefotaxime was initiated. However, the lack of clinical improvement after 48 hours, persistent fever, intractable vomiting, and onset of tachypnea (46 breaths/min) prompted diagnostic reconsideration. Laboratory investigations demonstrated a marked inflammatory response (C-reactive protein 266 mg/L). Arterial blood gas analysis revealed metabolic acidosis (pH 7.33, PaCO_2_ 11 mmHg, HCO_3_- 11 mmol/L) without hyperlactatemia (lactate 1.6 mmol/L). Serum electrolytes showed hyponatremia (129 mmol/L) and hypokalemia (3.2 mmol/L), with preserved renal function. Given the acute tachypnea, pulmonary embolism was suspected, and CT pulmonary angiography was performed, excluding this diagnosis. Alternative diagnoses were systematically considered and ruled out, including appendicitis, cholecystitis, pancreatitis, renal infarction, adrenal hemorrhage, Hemolysis, Elevated Liver Enzymes and Low Platelets (HELLP) syndrome, and acute fatty liver of pregnancy. Because magnetic resonance imaging (MRI) was not emergently available at our institution and given the persistence of unexplained symptoms, contrast-enhanced abdominal CT was performed, revealing bilateral adrenal enlargement without contrast enhancement (Figure 1, Figure 2). Both adrenal glands appeared enlarged with no parenchymal enhancement after intravenous contrast administration, consistent with adrenal ischemia and infarction. The coronal reconstruction confirmed the bilateral and symmetrical nature of adrenal involvement. Dotted lines on imaging highlighted the non-enhancing adrenal glands. Endocrine evaluation showed a serum cortisol level of 128.2 nmol/L, which is below the threshold suggestive of adrenal insufficiency in critically ill patients according to current guidelines. Adrenocorticotropic hormone (ACTH) measurement and dynamic testing were not immediately available and were not performed to avoid delaying urgent treatment.

**Diagnosis:** the final diagnosis was bilateral adrenal infarction complicated by acute adrenal insufficiency. This diagnosis was based on CT findings of bilateral adrenal enlargement without contrast enhancement, associated with an inappropriately low cortisol level in the context of severe physiological stress. The radiological evidence of complete bilateral adrenal involvement provided a strong anatomical basis for adrenal failure.

**Therapeutic interventions:** intravenous hydrocortisone was initiated immediately at a total daily dose of 200 mg, followed by gradual tapering. Isotonic saline infusion was administered to correct hyponatremia. Given the suspected thrombotic mechanism, therapeutic anticoagulation with enoxaparin was started. A multidisciplinary decision favored continuation of anticoagulation throughout pregnancy and planned vaginal delivery. The patient was instructed to discontinue enoxaparin at the onset of labor to allow a 24-hour window for safe neuraxial anesthesia.

**Follow-up and outcome of interventions:** the patient showed rapid clinical and biochemical improvement following corticosteroid therapy, with resolution of metabolic acidosis, decrease in inflammatory markers, and normalization of respiratory parameters, allowing transfer back to the obstetrics department on day five. At 38 weeks of gestation, she presented with premature rupture of membranes; enoxaparin was discontinued 24 hours before induction, epidural analgesia was safely performed, and vaginal delivery occurred under stress-dose hydrocortisone with favorable maternal and neonatal outcomes. A comprehensive thrombophilia work-up was performed at 2.5 months postpartum. Results showed normal antithrombin (98%) and protein C (93%) activities, with moderately decreased free protein S (58.4%). Activated protein C resistance (APCR) was detected without the factor V Leiden mutation. Genetic analysis revealed no factor V Leiden, R2 haplotype, Y1702C variant, or factor II G20210A mutation. A heterozygous methylenetetrahydrofolate reductase (MTHFR) C677T mutation was found without the A1298C mutation. Immunological work-up showed negative antinuclear antibodies, negative anticardiolipin antibodies (IgG/IgM), and isolated positive anti-β2 glycoprotein I IgM (27.6 RU/mL) with negative lupus anticoagulant. A repeat immunological panel at 3 months showed complete negativity of anti-β2 glycoprotein I antibodies, ruling out antiphospholipid syndrome according to international criteria. The APCR without factor V Leiden mutation was considered an acquired phenomenon related to peripartum hemostatic changes. The heterozygous MTHFR mutation has limited clinical significance in the absence of documented hyperhomocysteinemia. No major inherited thrombophilia was identified.

**Patient perspective:** the patient reported significant relief of symptoms within 24 hours after initiation of hydrocortisone therapy. She expressed satisfaction with the multidisciplinary care provided throughout hospitalization and delivery. She was reassured by the successful vaginal delivery and the favorable outcome for both herself and her newborn.

**Informed consent:** written informed consent was obtained from the patient for the publication of this case report, including the accompanying images.

## Discussion

Bilateral adrenal infarction during pregnancy is an uncommon condition whose recognition has increased with improved imaging access. It primarily results from adrenal vein thrombosis favored by pregnancy-induced hypercoagulability [1,2]. Physiological changes during gestation, including elevated procoagulant factors, reduced fibrinolysis, and venous stasis, significantly increase the risk of thrombotic events, predisposing the adrenal glands to ischemia even in the absence of underlying constitutional thrombophilia [2]. The diagnosis remains exceptionally challenging due to the nonspecific nature of its clinical presentation. Patients often present with acute abdominal or flank pain, fever, and vomiting, which overlap with more frequent conditions such as acute pyelonephritis, pulmonary embolism, acute surgical abdomen (appendicitis, cholecystitis), or renal infarction [3]. In this case, the initial suspicion of pyelonephritis was supported by lumbar tenderness, fever, and leukocyturia. However, the absence of microbiological confirmation, the lack of clinical response to appropriate antibiotic therapy, and the emergence of unexplained tachypnea and metabolic acidosis were key indicators that prompted further investigation. The systematic exclusion of obstetric (placental abruption, HELLP syndrome, acute fatty liver of pregnancy), surgical (appendicitis, cholecystitis, pancreatitis), and vascular (renal infarction, mesenteric ischemia, pulmonary embolism) alternatives strengthened the diagnostic pathway. Imaging is the cornerstone of the diagnostic process. While MRI is the preferred modality during pregnancy to avoid fetal ionizing radiation, contrast-enhanced CT remains a critical tool in emergency settings where rapid diagnosis is required and MRI is unavailable [5,6]

In our case, computed tomography pulmonary angiogram (CTPA) was already indicated to rule out pulmonary embolism, and the same contrast-enhanced examination simultaneously evaluated the adrenals without additional radiation. The estimated fetal radiation dose from abdominopelvic CT is < 10 mGy, well below the 50-100 mGy threshold associated with fetal harm. The pathognomonic radiological finding is bilateral adrenal enlargement with a lack of contrast enhancement, reflecting adrenal ischemia and infarction [5,6]. From a critical care perspective, the early identification of adrenal insufficiency is vital. The decision to initiate immediate hydrocortisone therapy was double-grounded: first, on the biochemical evidence of an inappropriately low cortisol level (128.2 nmol/L) for a patient under maximal physiological stress (pH 7.33, tachypnea 46/min, fever); and second, on the radiological proof of complete bilateral adrenal destruction. In cases of adrenal vein thrombosis, bilateral involvement implies a complete loss of functional adrenal cortex, making clinical adrenal crisis an anatomical certainty [4]. This proactive management, guided by multidisciplinary endocrinological assessment, is essential to prevent the rapid onset of a fatal adrenal crisis during the acute phase or the subsequent stress of labor. Concomitant correction of electrolyte imbalances, particularly hyponatremia, is also fundamental to stabilization. The etiological work-up in our patient did not reveal major inherited thrombophilia, suggesting that pregnancy-related hypercoagulability was the primary driver. This aligns with literature reporting BAI in otherwise healthy pregnant women [7,8]. The transient activated protein C resistance observed here likely represents an acquired phenomenon, potentially related to elevated factor VIII levels during the peripartum period. The isolated anti-β2 glycoprotein I IgM positivity that normalized on repeat testing did not meet criteria for antiphospholipid syndrome. The heterozygous MTHFR mutation has no established clinical significance without hyperhomocysteinemia.

Compared with previously reported cases of bilateral adrenal infarction in pregnancy, this case offers several distinctive features: i) it explicitly outlines a stepwise diagnostic pathway from suspected pyelonephritis to adrenal infarction, highlighting discordant clinical clues; ii) it provides a complete thrombophilia work-up with negative findings, reinforcing pregnancy-related hypercoagulability as the primary mechanism; iii) it demonstrates successful vaginal delivery under epidural analgesia with precise timing of anticoagulation discontinuation and stress-dose corticosteroids; iv) it is one of the few reported cases from the African continent, where emergency MRI may be unavailable, justifying a CT-first approach in life-threatening situations. The anesthetic and obstetric management required a delicate balance between thrombotic and hemorrhagic risks. Our preference for vaginal delivery in this primiparous patient was based on minimizing surgical trauma and potential retroperitoneal or operative site bleeding. The key challenge was managing the 24-hour window required between the last therapeutic dose of low-molecular-weight heparin and epidural catheter placement to prevent spinal hematoma. Our strategy of patient education and expectant management, followed by induction after premature rupture of membranes, allowed for a controlled 24-hour anticoagulation-free interval, ensuring safe neuraxial anesthesia and successful delivery under stress-dose hydrocortisone coverage. This approach aligns with American College of Obstetrics and Gynecology (ACOG) and Royal College of Obstetricians and Gynaecologists (RCOG) recommendations for managing therapeutic anticoagulation in late pregnancy. We acknowledge that an elective cesarean section could have been scheduled, but this would have exposed the patient to higher surgical bleeding risks without clear benefit in this stable primiparous patient. Strengths of this report include a detailed clinical course, comprehensive diagnostic evaluation with systematic exclusion of differential diagnoses, complete thrombophilia work-up, and explicit discussion of management trade-offs. Limitations include the use of CT instead of MRI (justified by clinical urgency and MRI unavailability), the absence of ACTH measurement or dynamic testing (not available emergently), and the lack of long-term follow-up beyond the postpartum period.

## Conclusion

This case reports a bilateral adrenal infarction in late pregnancy initially misdiagnosed as acute pyelonephritis, revealed by persistent symptoms and confirmed by contrast-enhanced CT, leading to acute adrenal insufficiency successfully managed with corticosteroids and anticoagulation. Bilateral adrenal infarction should be considered in pregnant patients with persistent unexplained abdominal or flank pain unresponsive to initial therapy, as early imaging and prompt corticosteroid replacement are crucial to prevent life-threatening adrenal crisis.
